# Complete genome sequence of bacteriophage Byron23 targeting *Microbacterium foliorum*

**DOI:** 10.17912/micropub.biology.001805

**Published:** 2025-12-08

**Authors:** Francisco J. Moro, M. Victoria Vallejos, Gina Cervesato, Ernestina Miniussi, Nicolás Cantero, Marilina Gaffuri, Milagros García, Zoe Clementino, Malena Quadri, Francisco Lamagni, Tulio Pianesi, Hugo Gramajo, Laura Buttigliero, Corina M. Fusari, Martín Espariz, María Alejandra Mussi, Lautaro Diacovich

**Affiliations:** 1 Facultad de Ciencias Bioquímicas y Farmacéuticas. Universidad Nacional de Rosario. Argentina; 2 Instituto de Física Rosario (IFIR-CONICET-UNR) Argentina

## Abstract

In this paper, we report the discovery and characterization of Byron23, a bacteriophage isolated from a soil sample collected approximately 30 cm beneath the surface of a stream bank in Rosario, Santa Fe, Argentina. Byron23 infected the host *Microbacterium foliorum* NRRL B-24224 resulting in clear, round plaques surrounded by a turbid edge. Transmission electron microscopy revealed that Byron23 exhibits siphoviral morphology. Genome sequencing using the Illumina platform revealed a 41,821 bp genome encoding 64 predicted genes. The bacteriophage was classified within the subcluster EA1 and is predicted to be a lytic phage.

**Figure 1. Characterization of the Microbacterium bacteriophage Byron23:**
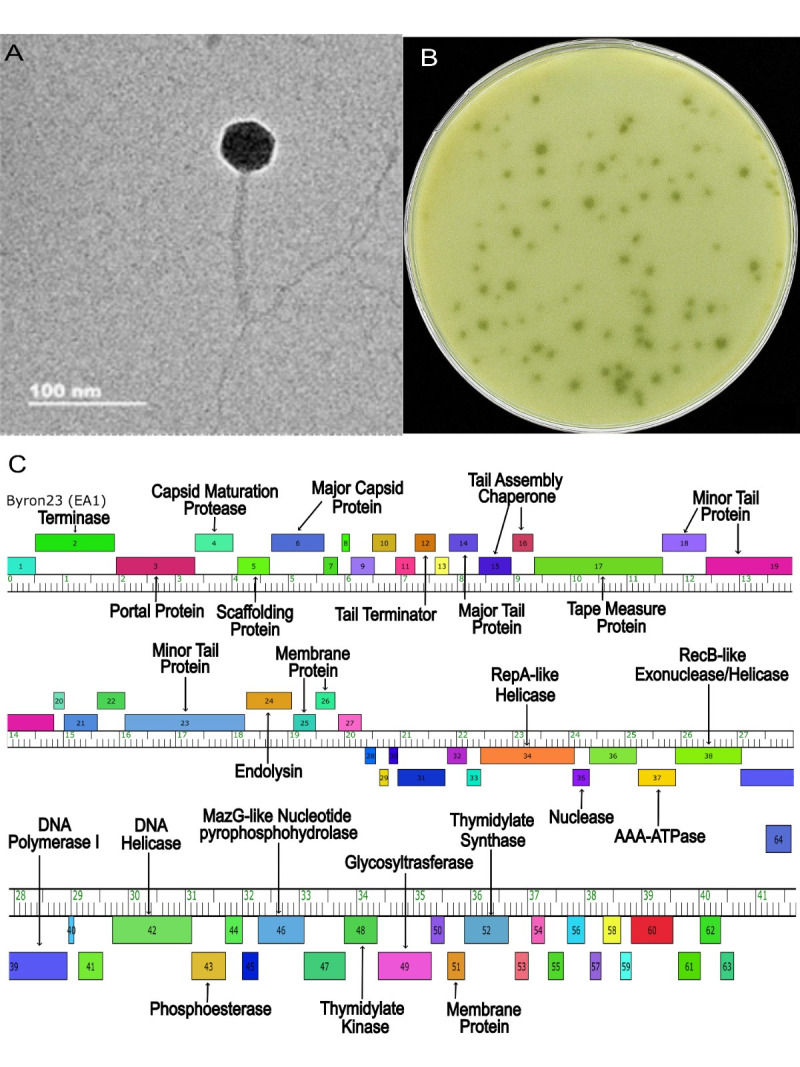
(A) Negative-stain (1% uranyl acetate) transmission electron microscopy revealed a siphoviral morphology; an icosahedral capsid of 47 nm ± 1 nm and a tail length of 122 nm ± 2 nm (n = 3). Scale bar = 100 nm. Image produced by a Zeiss Libra 120 TEM with an accelerating voltage of 80 kV and processed on Olympus Soft Imaging GmbH. 5.0 (Build 1194). (B) Byron23 forms plaques ranging from 2 to 4 mm in diameter, each surrounded by a turbid edge. (C) Genome organization of Byron23 as visualized using Phamerator. Genes are represented by boxes located above or below the ruler, depending on their forward or reverse orientation, respectively. Where applicable, presumed functions of the proteins are shown.

## Description

Bacteriophages are abundant and genetically diverse viruses that specifically infect bacterial hosts. Their relevance in medicine and biotechnology has increased in recent years due to their potential applications in combating antibiotic resistance (Kakasis and Panitsa, 2019), as well as their utility in genetic engineering (Sharma et al., 2017). In this work, we report the isolation and characterization of Byron23, a phage isolated on *Microbacterium foliorum* .

&nbsp;

Byron23 was isolated from a moist soil sample collected approximately 30 cm beneath the surface of the banks of the Ludueña Stream in the Funes/Fisherton area, a location within Rosario, Argentina (GPS coordinates 32.919765º S, 60.757799º W), using standard methods (Zorawik et al., 2024). Briefly, the soil sample was suspended in PYCa liquid medium and incubated at 28° C for 1 hour with gentle shaking at 250 rpm. The mixture was then centrifuged at 3500 rpm and filtered through a 0.22 µm syringe filter. The resulting filtrate was mixed with *Microbacterium foliorum* NRRL B-24224 and plated on PYCa soft agar before being incubated at 28 °C for 48 hours (Zorawik et al., 2024). Multiple plaques were observed; one plaque, characterized by a clear center and turbid edge, was selected for further purification ( Fig. 1B ). The selected plaque was purified through three successive rounds of plating; the minimum distance of the picked plaque was two centimeters. A high-titer lysate (7 x 10^8 PFU/ml) was subsequently prepared by flooding a plate with a very high density of plaques. To examine phage morphology via transmission electron microscopy (TEM), phage particles from a high-titer lysate were absorbed onto a carbon-coated copper grid, then stained with 1% uranyl acetate.&nbsp; Measurements of phage particles (n=3) revealed a head diameter of 47 nm ± 1 nm and a tail length of 122 nm ± 2 nm, consistent with siphoviral morphology ( Fig. 1A ).

&nbsp;

Genomic DNA was extracted from high-titer lysate using Monarch PCR and DNA cleanup kit (New England Biolabs), following the manufacturer's instructions. The genome was sequenced using an Illumina MiSeq sequencer (v3 reagents). The library was prepared using the NEB Ultra II Library kit, yielding 409,940 single-end 150-bp reads, which produced 1,401-fold coverage. Raw reads were assembled and checked for completeness using Newbler v2.9 (Miller et al., 2010) with default parameters and Consed v29 (Gordon and Green, 2013), respectively, as described previously. DNA concentration and purity were assessed using a NanoDrop spectrophotometer, and DNA integrity was confirmed by agarose gel electrophoresis. The genome is circularly arranged and contains 41,821 base pairs. It was bioinformatically linearized, determining base 1 by comparison to other phages in the database as described (Russell 2018).

&nbsp;

The Byron23 genome was annotated automatically using DNA master v5.23.6 (Pope and Jacobs-Sera 2018), Glimmer v3.02b (Delcher et al., 2007), and GeneMark v2.5p (Besemer and Borodovsky 2005), with start codon predictions manually refined using Starterator v 1.2, database v508 ( http://phages.wustl.edu/starterator/ ) and Phamerator Actino_draft v578 (Cresawn et al., 2011).

Based on gene content similarity of 63.6% with known phages, Byron23 was assigned to cluster EA1 according to the Actinobacteriophage database, phagesdb (https://phageDB.org) (Pope et al., 2017; Russell and Hatfull, 2017). The genome was bioinformatically linearized, determining base 1 by comparison to other phages in the database as described. Sixty-four putative protein-encoding genes were identified, and 25 of them could be functionally annotated using BLASTP v2.14.0 (against PhagesDB and NCBI non-redundant databases) (Altschul et al., 1990) and HHPred (with reference to the PDB mmCIF70, Pfam-v.36, and NCBI Conserved Domain databases) (Söding et al., 2005). Aragorn v1.2.41 did not detect tRNA genes in the Byron23 genome (Laslett and Canback 2004). All software tools were executed using default parameters.

One region of the genome encodes proteins involved in virion structure and assembly, including major and minor tail proteins, capsid maturation proteases, tape measure proteins, major capsid protein, tail assembly chaperone, and endolysins. The other region contains genes related to DNA metabolism, such as DNA recombinases, DNA helicases, DNA polymerase I, and phosphatases ( Fig. 1C ). Consistent with the predicted lytic lifecycle of EA1 subcluster phages, Byron23 lacks identifiable immunity repressor or integrase functions. Also, the majority of Byron23's predicted genes have no known function and are transcribed in the reverse direction (Bendele et al., 2025). Turbid edges around plaques were observed during infection, indicating potential depolymerase activity (Yan et al., 2014). However, genome annotation did not reveal any gene with a known depolymerase function. Further analysis is required to elucidate the genetic basis of this phenotype.

**&nbsp;**

**NUCLEOTIDE SEQUENCE ACCESSION NUMBERS**

Byron23 is available at GenBank with Accession No. PQ362663 .1 and the SRA accession No. SRX28943189 .

&nbsp;
